# THERAPY OF ENDOCRINE DISEASE: Perspectives on the management of adrenal insufficiency: clinical insights from across Europe

**DOI:** 10.1530/EJE-13-0450

**Published:** 2013-12

**Authors:** Ashley Grossman, Gudmundur Johannsson, Marcus Quinkler, Pierre Zelissen

**Affiliations:** 1 Oxford Centre for Diabetes, Endocrinology and Metabolism, Churchill Hospital, University of OxfordHeadington, Oxford, OX3 7EJUK; 2 Department of EndocrinologyInstitute of Medicine, Sahlgrenska Academy, University of GothenburgGothenburgSweden; 3 Clinical Endocrinology, Charité Campus MitteCharité University MedicineBerlinGermany; 4 Section of Endocrinology, Department of Internal MedicineUtrecht University Medical CenterUtrechtThe Netherlands

## Abstract

**Background:**

Conventional glucocorticoid (GC) replacement for patients with adrenal insufficiency (AI) is inadequate. Patients with AI continue to have increased mortality and morbidity and compromised quality of life despite treatment and monitoring.

**Objectives:**

i) To review current management of AI and the unmet medical need based on literature and treatment experience and ii) to offer practical advice for managing AI in specific clinical situations.

**Methods:**

The review considers the most urgent questions endocrinologists face in managing AI and presents generalised patient cases with suggested strategies for treatment.

**Results:**

Optimisation and individualisation of GC replacement remain a challenge because available therapies do not mimic physiological cortisol patterns. While increased mortality and morbidity appear related to inadequate GC replacement, there are no objective measures to guide dose selection and optimisation. Physicians must rely on experience to recognise the clinical signs, which are not unique to AI, of inadequate treatment. The increased demand for corticosteroids during periods of stress can result in a life-threatening adrenal crisis (AC) in a patient with AI. Education is paramount for patients and their caregivers to anticipate, recognise and provide proper early treatment to prevent or reduce the occurrence of ACs.

**Conclusions:**

This review highlights and offers suggestions to address the challenges endocrinologists encounter in treating patients with AI. New preparations are being developed to better mimic normal physiological cortisol levels with convenient, once-daily dosing which may improve treatment outcomes.

## Introduction

If left untreated, adrenal insufficiency (AI) is a lethal condition and before the availability of glucocorticoids (GCs), the majority of patients with primary AI died within 2 years of diagnosis (1).

For several years, the life expectancy of patients with AI who received conventional GC replacement therapy and adequate follow-up was considered normal (2, 3). However, it has recently been determined that patients with primary AI have a more than twofold increased standardised mortality ratio (SMR) mainly due to cardiovascular and infectious diseases (4, 5). Similarly, patients with secondary AI have an increased mortality rate mainly due to cardiovascular disease (6, 7). Recent data have demonstrated that the metabolic cardiovascular risk profile in patients with hypopituitarism and AI is related to the daily dose of hydrocortisone (HC) (8). In addition to decreased life expectancy, patients with AI on current replacement therapy also have a significantly compromised quality of life (QoL), irrespective of the origin of AI or concomitant disease (9, 10).

In healthy individuals cortisol secretion follows a circadian rhythm (11, 12). Conventional GC replacement therapy in patients with AI does not provide appropriate physiological replacement in terms of precisely mimicking this rhythm (13). Over-replacement of GC may lead to morbidity including impaired glucose tolerance, obesity, bone metabolism, osteoporosis and sleep disturbance (14). In particular, overexposure during parts of the day when cortisol exposure is normally low may lead to symptoms and signs related to overexposure (15). Conversely, under-replacement can result in significant impairment of well-being and can be life threatening if the patient develops an intercurrent illness.

The challenge of management is to tailor the GC replacement therapy to the needs of each patient. To prevent adrenal crisis (AC) there are three areas to be considered: i) the daily maintenance dose, ii) the daily dose/exposure of cortisol required to mimic normal physiology and iii) the need for extra cortisol during an intercurrent illness or non-illness-related events, such as exercise, in order to prevent AC.

This review considers the current management of AI and unmet medical needs based on the literature and our experience of treating patients with AI. We provide practical advice for endocrinologists and other physicians who face the challenges of managing AI in diverse situations.

## What are the considerations for achieving short- and long-term goals of AI treatment?

**Figure 1:**
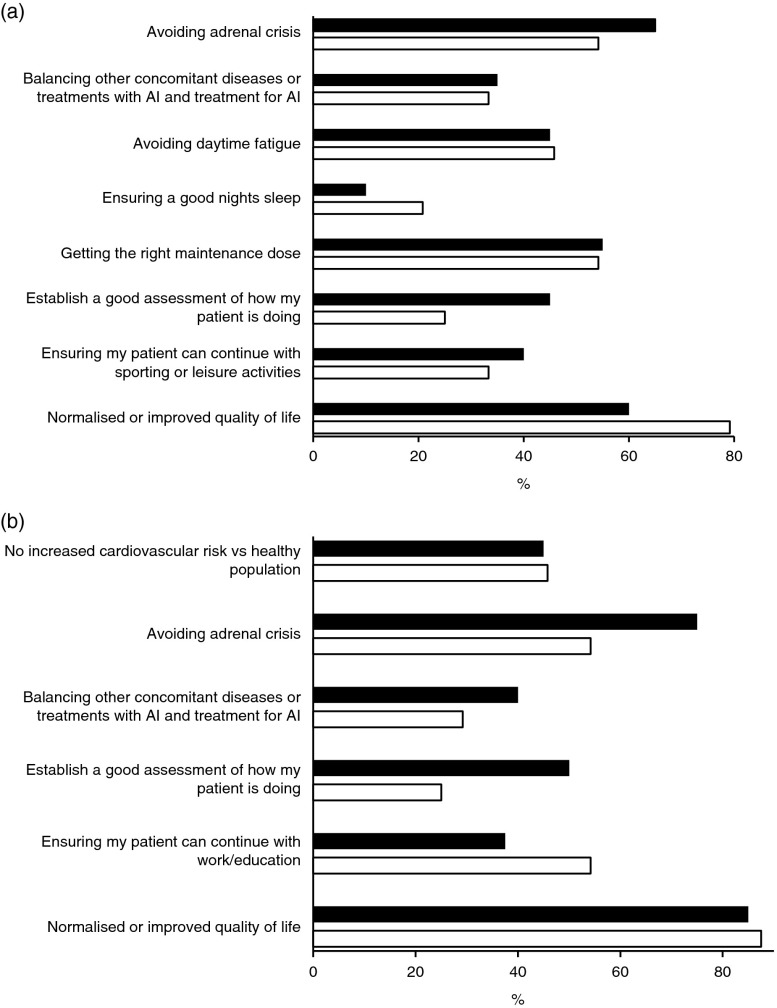
Results of an electronic survey regarding (a) short-term and (b) long-term goals in the treatment of patients with adrenal insufficiency among the participants of the German Endocrine Society Meeting in March 2013 (black bars) and of the British Endocrine Society in March 2013 (white bars). Participants (28 from Germany and 36 from UK) included endocrinologists (54.6 and 69.4%), other clinicians (10.7 and 5.6%), researchers (10.7 and 11.1%), nurses (7.1 and 8.3%) and others (17.9 and 5.6%), respectively.

An electronic survey sponsored by ViroPharma (SPRL, Brussels, Belgium) was distributed by the learned endocrine societies of Great Britain and Germany to the participants of the German Endocrine Society Meeting and of the British Endocrine Society both in March 2013. The three most important short-term goals of AI treatment identified were the same in the UK and Germany (Fig. 1a): i) to identify the optimal GC dosing regimen to restore/mimic the normal physiological cortisol circadian rhythm, ii) to improve patient QoL and iii) to prevent AC by responding to the increased need for cortisol during intercurrent illness and mental or physical stress.

Achieving these short-term goals is difficult because GC needs can change during the day as well as during times of physiological and psychological stress. Developing GC therapy to perfectly parallel the physiological cortisol circadian rhythm remains a challenge (13, 16, 17, 18).

Furthermore, monitoring of replacement therapy to optimise the individual replacement dose or make dose adjustments is largely based on clinical signs and symptoms, rather than objective biological serum markers assessing the tissue activity of cortisol. Furthermore, the signs and symptoms indicating overtreatment (i.e. weight gain and skin alterations) or under-treatment (i.e. fatigue, nausea, myalgia and joint stiffness) (13, 14, 19, 20) are not specific to AI. Some patients exhibit a normal basal cortisol level but have a subnormal response to stimulation (21). Such patients may need a regular but smaller dose of HC while others require a small dose of HC to be available for emergency use. This is a difficult situation, as no normative levels have been published and it is a matter of fine clinical judgement as to how to handle such relatively common situations. The minimum requirements are probably to make available a supply of GC for emergency use and to ensure that the patient is aware of emergency measures; however, small additional doses of HC are very much a question of individual judgement.

The top three long-term goals were also similar between Germany and UK participants in the previously mentioned survey (Fig. 1b) and in general agreement with other studies. The long-term goals of lifelong GC replacement are to i) improve QoL, ii) avoid or anticipate AC, iii) avoid the dangers of over-replacement, specifically associated with metabolic syndromes, cardiovascular disease and osteoporosis, and iv) normalise the SMR (8, 22, 23).

## What are the challenges associated with conventional treatments for AI?

None of the conventional GC treatments can perfectly imitate the physiological cortisol rhythm. The fall in cortisol around midnight seems to be especially important to guarantee normal physiology. GC administration in the evening seems to predispose individuals to metabolic changes and sleep disorders. Interestingly, a lesser decrease in cortisol is observed in patients with depression. In addition, increased cortisol levels at night are significantly associated with light sleep and wakefulness (24, 25). The early morning cortisol rise before waking is regarded as important (12, 23), although only a few studies have addressed this topic (26, 27).

**Figure 2:**
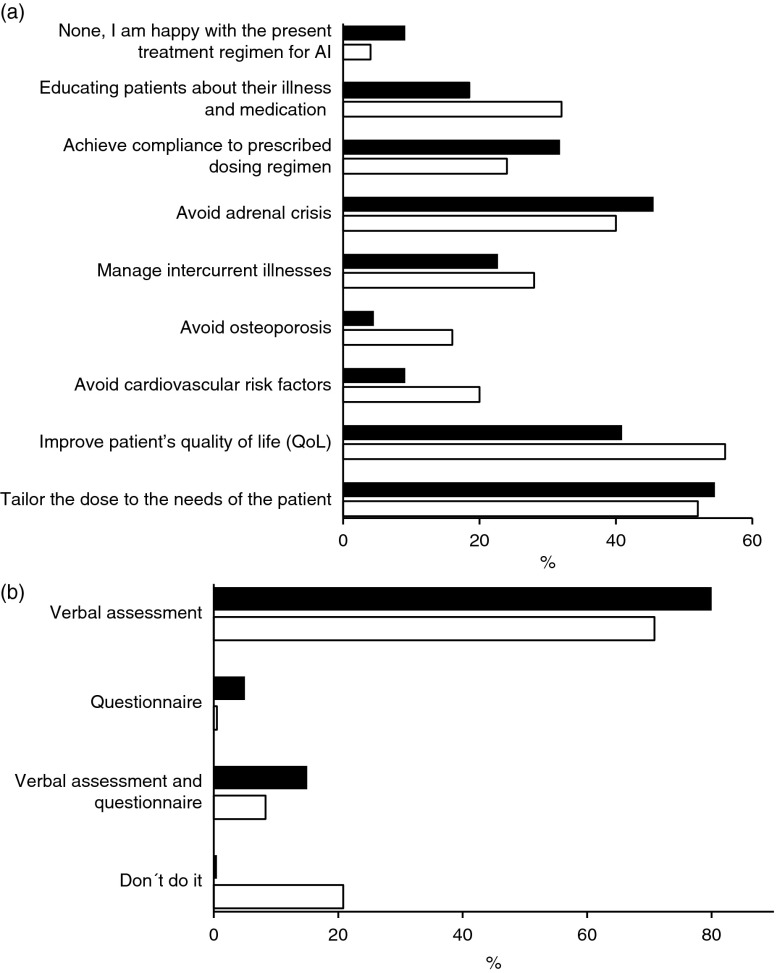
Results of an electronic survey among the participants of the German Endocrine Society Meeting in March 2013 (black bars) and of the British Endocrine Society in March 2013 (white bars) regarding (a) the three main challenges with the present treatment regimens of conventional therapy and (b) the current assessment of well-being and quality of life in patients with adrenal insufficiency.

The main challenges with conventional treatments are shown in Fig. 2a. A major aspect is the actual daily dose that may be too high, as well as an unphysiological exposure pattern that provides high exposure late during the afternoon and evening (Box 1). Weight-related dosing of HC can reduce GC overexposure but still does not replicate the normal cortisol rhythm (13, 16). Determination of the optimal daily dose, cortisol-time exposure and modification of treatment to accommodate a patient's response to stress and minor illness also present challenges. There is no target to titrate against in AI, and dose determination is generally based on cortisol production in an average healthy person and the individual patient's response. Interestingly, our survey showed that QoL is both a major goal and main challenge; however, it is still not routinely assessed by physicians (Fig. 2b).

HC is the most commonly used GC for AI therapy. Dose replacement with HC should be customised on an individual basis and may be guided by weight-related regimens. HC has high oral bioavailability, but has a short half-life between 60 and 120 min (28). The serum cortisol profile resulting from current GC replacement therapies exhibits steep peaks followed by a rapid decline to trough cortisol levels with little resemblance to the normal physiological circadian pattern. Current treatment practice requires that the total daily dose of immediate-release HC should be divided and administered two or ideally three times daily (Table 1). However, even when HC is administered multiple times per day the serum cortisol profile is still far from paralleling the normal physiological cortisol circadian rhythm (13, 16, 18).

Administration of multiple daily doses of HC poses several problems, in particular during GC replacement. There are peaks and troughs resulting in overexposure followed by underexposure to GC. Also, the timing of the GC dose is likely to be different on a day-to-day basis resulting in different time-exposure profiles. Consequently, patients with AI on replacement therapy report impaired health-related QoL and often experience fatigue during the day before their next HC dose. Such patients report subsequent socioeconomic health problems such as hospitalisation, absenteeism from work and need for disability pensions (9, 10, 13, 29). In an international survey of 1245 patients with primary and secondary AI, multiple daily dosing was reported as a problem by 38% of patients. Patients who reported difficulties with multiple dosing also reported greater fatigue and impact on QoL (9). Forgotten doses can also pose an increased risk for AC.

In small, uncontrolled studies in patients with AI, HC i.v. infusions by a programmable pump have been reported to restore a normal cortisol circadian rhythm and improve all subscales of QoL. However, HC infusions are cumbersome and clearly not practical in general clinical practice. In addition, metabolic and QoL outcomes resulting from infusion treatments have not been systematically reported (11, 22, 30).

In summary, the non-physiological circadian profile is believed to be the major explanation for worse outcome of AI, although the level of evidence for this is still poor due a lack of randomised, placebo-controlled studies. Other issues such as actual dose and management of interrcurrent illness can negatively impact outcomes.

In some European countries, cortisone acetate is used as GC replacement. Cortisone acetate can be administered up to three times daily (Table 1). It requires conversion to HC via the hepatic enzyme 11β-hydroxysteroid dehydrogenase type 1 *in vivo* and, as such, has a lower cortisol peak, slower onset of action and slower decline to trough than HC (16, 29). However, the activity of cortisone acetate may be affected by inhibition of the conversion enzyme (31).

Prednisolone, administered as a single morning dose, has also been used as GC replacement (Table 1) (16). Prednisolone has more sustained action compared with HC (12–36 vs 6–10 h) (16). Owing to its higher potency (four to five times higher than HC), the once-daily dose should range between 3 and 5 mg. Dexamethasone has an even longer half-life of 36–72 h, but is not routinely used to treat AI (Table 1) (13, 16). There is a risk of increased long-term adverse effects, such as osteoporosis, due to the long duration of action of prednisolone and dexamethasone (13, 32). Furthermore, and most importantly, unlike HC or cortisone acetate, it is not possible to model replacement in terms of measurement of blood or saliva levels thereby limiting the customisation of dose scheduling. A study examining the differences in health status related to the use of HC, cortisone acetate or prednisolone treatment in patients with AI failed to demonstrate any meaningful differences in health-related QoL based on the type of GC used for replacement therapy (16, 33). Experimental studies have also demonstrated that the GC interaction/binding with the GC receptor is very different with long-acting synthetic steroids as compared with HC (34). The importance of this for the replacement situation is not known. Consequently, no current regimen of replacement therapy is ideal in terms of the avoidance of ACs, the risks of over-replacement and the normalisation of QoL.

## Should treatment be initiated in all patients diagnosed with AI?

### Will the patient need to take GC replacement medication for the rest of their life?

#### Primary AI

Primary AI is considered to be an incurable disease with a need for lifelong GC (and mineralocorticoid) replacement therapy. Apart from one exceptional case in which partial recovery of autoimmune Addison's disease primary AI was documented (35), no adrenocortical recovery, determined by adrenocorticotropic hormone (ACTH) stimulation tests, was found in 27 patients with autoimmune Addison's disease (36).

#### Secondary AI

Many patients with hypopituitarism develop secondary AI (20). Secondary AI (as part of hypopituitarism caused by large pituitary adenomas) has been reported to be reversible after extirpation of the adenoma (37, 38, 39), although that is not the case in all studies (40). Secondary AI caused by a macroprolactinoma has also been reported to be reversible after shrinkage of the tumour by dopaminergic medication in some but not all studies (41). As many patients with hypopituitarism marginally fail diagnostic testing for AI (21), it remains a challenge to determine the initial dose for replacement therapy in an individual patient.

#### Temporary AI

Cushing's syndrome caused by a corticotroph pituitary adenoma (Cushing's disease) or by ectopic ACTH secretion leads to AI after successful treatment of the source of excessive ACTH secretion. This is caused by suppression of normal pituitary corticotroph cells as a result of long-term hypercortisolaemia. The AI in this particular situation can last for several months or years until full recovery of adrenocortical function occurs after gradually diminishing the dose of GC replacement. The same is true for the AI following unilateral adrenalectomy for a cortisol-producing adenoma or carcinoma.

#### Tertiary AI (excess exogenous GC)

Secondary AI caused by HPA axis suppression by long-term, high-dose, exogenous GC therapy (also called tertiary AI) is also reversible if gradual withdrawal of the exogenous steroids is feasible (42). These patients often need GC replacement only in emergency situations or during episodes of intercurrent illness and physical or severe mental stress. However, physicians should be aware that AI may become longstanding or even permanent in exceptional cases.

### When is DHEA treatment indicated in patients with AI?

DHEAS are androgen precursors secreted primarily by the adrenal cortex, which in peripheral tissues are converted to more potent androgens such as testosterone and oestrogens. In both primary and secondary AI, DHEA secretion is clearly decreased and often even absent in both men and women with hypoadrenalism. DHEA deficiency with reduced vitality and libido is clinically more evident in women due to the usually preserved gonadal androgen production in men (43, 44).

Apart from its mild androgenic effects, DHEA may also act as a neurosteroid with possible effects on mood, cognition and well-being. The first randomised controlled trial of DHEA in 24 female patients with primary or secondary AI showed that DHEA 50 mg daily for 4 months led to improvement in sexual function, decreased depression scores and improved well-being when compared with placebo (44). Further studies with DHEA replacement in patients with AI, however, showed conflicting results with regard to sexual function and QoL (45, 46), and in general any effects seen are usually relatively minor, at least in most patients.

The side effects of DHEA replacement include acne, hirsutism (although growth of the often sparse axillary and pubic hair may be a ‘desired’ effect by some female patients), alopecia, itching scalp/skin and particularly increased sweat odour when the dose is too high, which may occur more frequently in elderly women.

The dose of DHEA used in most studies is between 25 and 50 mg once daily. Clinical assessment of well-being, skin and axillary and pubic hair as well as plasma DHEAS trough levels and plasma testosterone can be measured as monitoring tools of the correct DHEA dose (23).

Based on the evidence currently available, DHEA replacement should not be undertaken routinely in clinical practice in patients with AI. Women with hypopituitarism with concomitant AI have a more severe androgen deficiency than those with primary AI. In this case, DHEA replacement could be tried on an individual basis for patients with persistent and seriously impaired QoL and reduced libido despite optimised conventional GC and mineralocorticoid replacement (Box 2) (19, 47). If no positive subjective effects are experienced by the patients after 3–6 months, it is probably best to discontinue the DHEA.

### Use of mineralocorticoids

#### Optimising mineralocorticoid dose

Under-replacement with mineralocorticoids has been reported in patients with primary AI (48, 49), but recent studies are lacking. It is conceivable that optimising (increasing the dose) mineralocorticoid replacement may facilitate lowering of the GC dose, although there are no reported data supporting this hypothesis. It is important to note that different GCs also have different mineralocorticoid activities, e.g. dexamethasone is devoid of any mineralocorticoid activity and prednisolone has less than HC. Thus, a patient with primary AI on dexamethasone would require a higher fludrocortisone dose than such a patient treated with HC.

#### Mineralocorticoid replacement in patients with primary AI and hypertension

In patients with primary AI who develop hypertension, the first step should be to assess possible mineralocorticoid (and also GC) over-replacement and lower the fludrocortisone dose. Even without clear mineralocorticoid over-replacement, the fludrocortisone dose could be mildly decreased while carefully monitoring signs of mineralocorticoid under-replacement.

#### No need for mineralococorticoid replacement in secondary AI

The secretion of aldosterone is primarily stimulated by the renin–angiotensin system with only a minor component under ACTH control. As a consequence, there is no need for mineralocorticoid replacement in secondary and tertiary AI.

### What treatment options exist for a patient with AI on HC 25–30 mg and still complaining of fatigue and loss of energy?

New estimates of the cortisol production rate in normal subjects indicate that a total dose of HC 15–20 mg is required daily (13, 23). The pharmacokinetic profile of immediate-release HC shows a steep rise to very high peak serum levels within 1–2 h of administration, followed by a rapid fall to very low levels 5–7 h after administration (23). Patients in whom HC is adequately replaced but who are still experiencing fatigue should be considered for multiple daily doses of HC. It is recommended that the total daily HC dose be divided into two or three doses to better simulate the physiological serum cortisol circadian rhythm (13, 23), with one-half to two-thirds of the daily dose administered in the morning and subsequent doses administered 5 h later (13). After a morning dose of HC, serum cortisol levels in the afternoon or evening will be low which may increase fatigue. An HC replacement dose should not be administered too close to bedtime because sleep disturbances may result due to high serum levels of HC (23). Other hormone replacement should be optimised, and changes in the blood levels of HC may follow initiation of thyroid hormone or growth hormone (GH) therapy. In particular, GH will decrease the activity of 11β-hydroxysteroid dehydrogenase type 1 leading to lower cortisol levels. Awareness and monitoring of these hormone interactions are important when determining optimal replacement therapy (50, 51). If fatigue and low energy continue, a once-daily modified-release GC replacement formulation could be introduced.

### What is an AC and when is it most likely to occur? What situations require an increase in GC replacement dose or an emergency injection of HC?

AC is a life-threatening complication in patients suffering from chronic AI and is usually defined as acute impairment of general health with the need for parenteral GC administration and probable hospital admission (19, 52, 53). ACs occur in AI with a frequency of about 6.3 per 100 patient-years (6.6 in primary AI and 5.8 in secondary AI) (52). Similar low but sustained rates of AC were also reported by Druce *et al.* (53a). The most common causes are mainly infection and fever (45%), but a number of other significant causes including surgery and pregnancy are also associated with AC (52). Patients with additional comorbidities are especially prone to crisis (53). It has been found that a major factor in the precipitation of AC is a lack of adequate education of the patient and their caregiver in terms of what actions to take in the event of an imminent AC. This also extends to a lack of appropriate action being taken by primary care physicians or even hospital attendants. Because infectious disease is one of the major causes of AC (54), it is essential that attending physicians treat any possible or suspected infectious disease aggressively and sufficiently increase the dose of GC (52).

#### Responding to an AC

All patients and their partners/caregivers should be educated such that they are able to recognise an imminent AC and understand how to adjust the dose of GC appropriately, including the use of parenteral GC (Box 3). While additional doses of HC do not seem to be needed for short-term physical activity, in cases of vigorous, sustained physical activity (e.g. playing a prolonged football game) and deep, long-lasting psychological stress (e.g. a bereavement), an additional HC dose of 5–10 mg is recommended and, ideally, should be given before the situation (19). It is essential to avoid over-replacement by resuming the lower standard dose as soon as possible after the crisis has passed. For more minor stressors, which include both physical and psychological stressors, such as dental procedures, it might be also wise to double the dose for 24 h. In case of illness and especially fever, the daily HC replacement dose should be doubled or tripled immediately to at least ∼30–60 mg/day (or even higher depending on the severity of the illness); however, there is a lack of an evidence base for this recommendation.

In general, it is important to cover the whole day by the increased dosing, and not to just double the dose in the morning. When modified-release HC (PLENADREN) is the replacement therapy, doubling or tripling the daily dose should be accomplished by taking the additional doses after 6–8 h. To complicate dose determinations, there is an apparent lack of dose proportionality that may be related to reduced drug availability or altered distribution with increasing dose (55). In situations where there is a major stressor, including major surgery, severe trauma and childbirth, and most importantly where there is diarrhoea/vomiting, HC should be administered intravenously or intramuscularly (100–400 mg/24 h).

#### Emergency cards and kits

**Figure 3:**
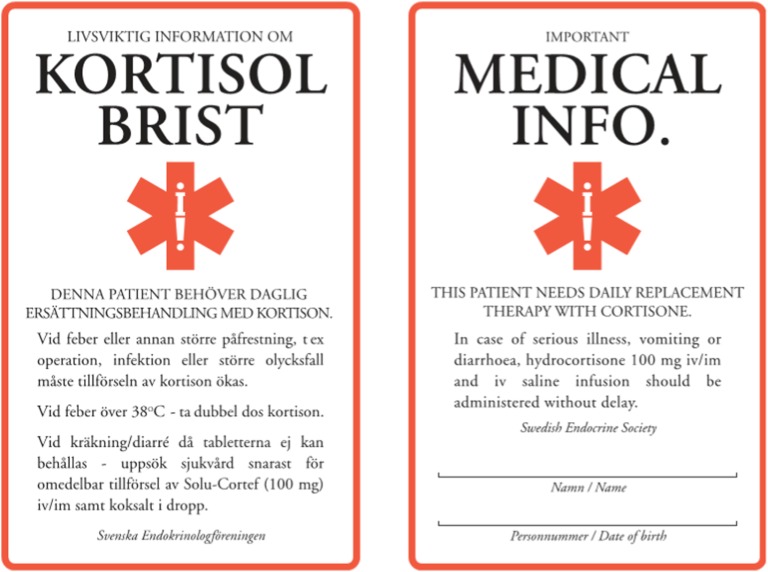
The Original Swedish Emergency Card was created by Dahlqvist *et al*. (56) in the Swedish Addison Registry and the Swedish Endocrine Society. This card was chosen for use among all other emergency cards used in Europe by an independent expert panel of European endocrinologists. Full colour version of this figure available via http://dx.doi.org/10.1530/EJE-13-0450.

The clinical features of an approaching AC may not be immediately recognised resulting in a delay of administration of emergency GC. We believe it is essential that all patients with AI should receive a ‘steroid emergency card’, which provides information as to the necessity for treatment, the current replacement regimen and any relevant contact information for the responsible clinician. The card should be recognised by other healthcare personnel such as paramedics and emergency responders. An example of an emergency card used in Sweden is shown in Fig. 3 (56). This card is conveniently sized to fit in a wallet and has the emergency information presented in Swedish on one side and English on the other. This card could easily be adapted for use in other countries.

All patients should also be provided with an emergency kit and be trained in the appropriate use of all components. This kit may contain:

- Rectal suppositories (prednisolone-suppository (Rectodelt 100)), which are easily administered. One suppository contains prednisolone 100 mg (equivalent to HC 400 mg). In the case of diarrhoea, rectal administration is not regarded as sufficient. HC suppositories (200 mg), which result in a peak in plasma HC levels between 1 and 2 h that persist for ≥8 h, have also been used to prevent ACs (57).
- An ampoule of 100 mg HC-21-hydrogensuccinate for i.m. or s.c. injection, injection devices and an instruction leaflet on self-administration in emergency situations (e.g. diarrhoea and vomiting) and in situations in which the increased oral HC dose failed to sufficiently improve the patient's symptoms. The patient or their regular caregiver or partner should be instructed in its use.

In one survey of patients with AI, 94% of respondents carried an emergency card, but only 30% possessed a GC emergency kit and 10% reported that they had never increased their GC dose (52). Only a few patients were able to or knew how to self-inject and most relied on medical personnel for emergency GC replacement treatment (53). Since many GC emergencies occur away from home (53), in addition to an emergency card, a GC emergency kit should be provided to every patient by the treating physician.

### How should AI be handled in special situations such as drug use with interfering medications or during pregnancy?

#### Interfering medications

**Figure 4:**
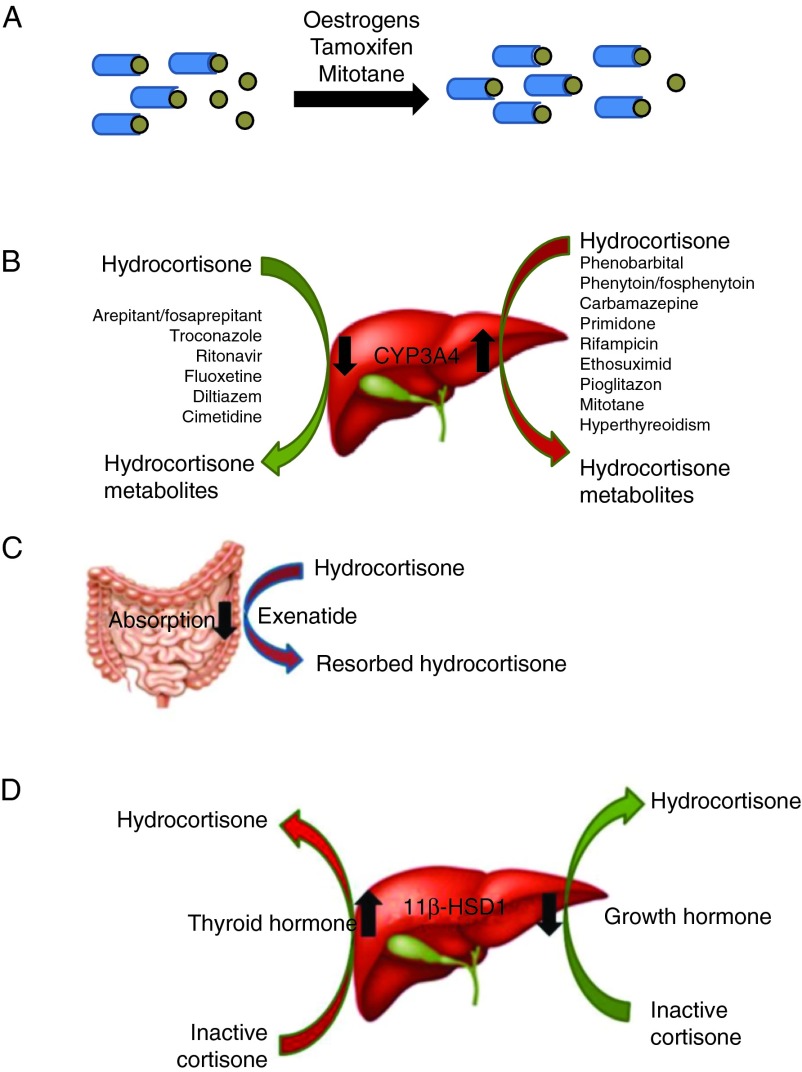
Interfering concomitant medications – mechanism of action. (A) Drugs inducing production of cortical-binding globulin might lower free unbound HC. (B) Drugs interfering with hepatic CYP3A4 influence HC metabolism causing GC under- or over-replacement. (C) Drugs might influence intestinal absorption of hydrocortisone. (D) Drugs modulating hepatic 11β-hydroxysteroid dehydrogenase type 1 (11β-HSD1) influence HC half-life. Full colour version of this figure available via http://dx.doi.org/10.1530/EJE-13-0450.

A number of drugs interfere with hepatic HC metabolism by influencing the CYP3A4 hydroxylating enzyme activity as illustrated in Fig. 4. HC doses must be adjusted accordingly when used concomitantly with interfering medications.

#### GC replacement during pregnancy

Women with known AI who become pregnant in most cases do not need adjustment of the GC replacement dose during the first two trimesters of pregnancy. An increased dose of HC (approximately by 20-50% of the initial dose) may be needed during the third trimester because of increased free cortisol during this period (19, 58).

In some pregnant women with primary AI, the mineralocorticoid dose may also need a small increase during the last trimester because of the anti-mineralocorticoid activity of progesterone (19). The pregnant patient with primary AI should be regularly monitored using blood pressure and plasma potassium rather than plasma renin as monitoring tools (58).

### What is new and what is ‘in the pipeline?’

New timed-release GC preparations may allow improved mimicking of the early morning cortisol rise before awakening in patients with AI and may hold the potential to positively affect QoL in patients with AI (59). In a recent preliminary study of seven patients treated for 3 months in an open-label design, the administration of HC via continuous infusion re-established the physiological circadian variation allowing reduction of the total GC dose and concomitantly improved subjective health status (30, 60). However, due to the complicated continuous infusion this therapy concept is not yet ready for widespread use.

A modified-released HC tablet, formerly known as Duocort (12, 26), is now available in some European countries under the brand name PLENADREN. It is administered in the morning and contains a rapid-release coating and a timed-released inner core of HC, suggesting that only a once-daily dose might be necessary to provide adequate coverage throughout the day. In the initial trial, PLENADREN showed an improvement in QoL, blood pressure and other metabolic parameters (61).

In a recent study in patients with rheumatoid arthritis, a timed-release prednisone tablet providing GC release before waking was able to markedly reduce disease activity when compared with a standard preparation of prednisolone at the same dose (62). This preparation (LODOTRA, available from Mundipharma, Cambridge, UK) is available for patients with rheumatoid arthritis and was recently tested in patients with AI (63). This modified-release prednisone showed decreased complaints and fatigue compared with standard prednisolone emphasising the importance of the GC increase in the early morning hours before waking (63). Prednisolone and LODOTRA may be useful options in a few exceptional cases.

#### Treatments in development

A new, oral, timed-released HC preparation (CHRONOCORT, available from Diurnal Limited, Cardiff, UK) imitates the physiological release of cortisol better than conventional preparations (59). When the tablet was given at 2200 h, cortisol levels remained low during midnight and increased 6–8 h later, reaching peak levels of 380 nmol/l at around 0600–0700 h. This modified-release formulation of HC which allows for delayed and sustained release has been shown to potentially replicate normal unstressed physiological cortisol levels (11).

## Summary and conclusions

Although immediate-release GC therapy has enabled patients with life-threatening AI to survive, management of AI continues to be challenging. Current therapies are inadequate and result in an increased mortality risk and compromised QoL. In addition, the failure of current treatment regimens to individualise total cortisol exposure and to mimic the normal physiological rhythm of cortisol release results in comorbidities such as cardiovascular disease and osteoporosis. Serum cortisol levels differ greatly between patients, and requirements change over the course of the day and in response to stressful situations and intercurrent illness. This variation necessitates the tailoring of therapy to individual patients and often results in the division of an overall GC dose into two, three or even four doses throughout the day. The need to take several doses each day interrupts activities and can be a reminder of illness, often resulting in poor patient adherence. Critically, patients, their relatives and caregivers must be taught to recognise signs of ACs and to be prepared to administer life-saving treatment. Hopefully, new HC preparations that are available and those in development will better mimic normal physiological cortisol levels with once-daily dosing and improve disease control and outcomes.

## Figures and Tables

**Table 1:** Features of available glucocorticoid, mineralocorticoid and androgen replacement (13, 16, 23, 47).

**Steroid**	**Features/challenges**	**Half-life** (h)	**Recommended total daily dose**^a^ (mg)	**Recommended dose frequency**	**Monitoring**
Glucocorticoid					
Hydrocortisone (=cortisol)	Physiological GC; 96% orally available; short half-life with steep peaks and troughs	1–2	20–25 for primary AI15–20 for secondary AI	Two or three doses with 1/2–2/3 of dose in the morning and subsequent doses later in the early afternoon/evening	No reliable and convenient marker to assess GC levels. Monitoring is based on clinical assessments indicating over- or under-treatment which are non-specific to AI
	Modified-release formulation (Plenadren)			Once in the morning	
Cortisone acetate	Lower serum cortisol peak; requires conversion to HC, which results in slow rise to peak and slower decline. May be affected by impairment of the enzyme 11β-hydroxysteroid dehydrogenase type 1 for conversion to HC		25–37.5	Once daily	
Prednisolone	Intermediate duration; more anti-inflammatory than mineralocorticoid	12–36	3–5^b^	Once in the morning	Prednisolone cross reacts in many cortisol assays
Dexamethasone	Mainly anti-inflammatory with no mineralocorticoid activity. Increased risk of excess exposure because of long half-life	36–72	Not recommended	Not recommended	Does not significantly cross-react in most assays
	Not used on a regular basis in AI therapy				
Mineralocorticoids					
9-α-Fludrocortisone	Selective binding to mineralocorticoid receptor		0.1 mg		
			Dose adjustments needed in hot climate, strong perspiration, pregnancy or in patients with concomitant hypertension	Once daily in the morning; or 1/2–0–1/2	Blood pressure, serum sodium and potassium, salt-craving, plasma renin activity/concentration with target at normal to upper normal reference range
Androgen					
DHEA	Not regarded as standard replacement regimen in AI		25–50	Once daily in the morning	Measure trough serum DHEAS concentrations, androstenedione, testosterone and sex hormone-binding globulin levels
	No pharmaceutical-licensed preparation available. Actual DHEA in over-the-counter preparations claiming 25 mg may vary from 0–140 mg				

a Dose equivalence calculations based on anti-inflammatory activity and may not be equivalent for cardiovascular effects; there is wide intra-individual and inter-individual sensitivity to GCs.

b Exact relative potency in humans is uncertain.

## **Box 1** Practical guidance to address a woman with weight gain for 3 years and tiredness.

**Table:**

**Case no. 1**	
Patient type/description	42-year-old-woman with Hashimoto's thyroiditis for 10 years, Addison's for 5 years
Management strategies	
Current medication	l-Thyroxine 75 μg/day, hydrocortisone 20 mg (0800 h)–10 mg (1400 h)–10 mg, fludrocortisone 0.1 mg 1–0–0
Current problems	Weight gain for more than 3 years; tiredness at midday and during the early evening
Therapeutic suggestions	HC dose reduction to 20 mg/day
	Split daily dose to three times daily (10–5–5 mg) or four times daily (7.5–5–5–2.5 mg) and start with first dose upon waking and last dose before 1800 h

## **Box 2** Practical guidance to address a female patient with Addison's disease.

**Table:**

**Case no. 2**	
Patient type/description	38-year-old woman, Addison's disease for 15 years
Management strategies	
Current medication	Hydrocortisone 10 mg (0700 h)–5 mg (1200 h)–5 mg (1700 h), fludrocortisone 0.1 mg 1–0–1/2
Current problems	No libido, loss of energy, stress intolerance at work
Therapeutic suggestions	Try DHEA 25 mg 1–0–0 for at least 6 months, adapt dose up to 50 mg according to plasma levels of androgens and side effects

## **Box 3** Practical guidance to address patient with Addison's disease and hypothyroidism and diabetes mellitus.

**Table:**

**Case no. 3**	
Patient type/description	42-year-old woman, hypothyroidism for 20 years, type 1 diabetes mellitus for 15 years, Addison's for 10 years
Management strategies	
Current medication	l-Thyroxine 62.5 μg/day, hydrocortisone 10 mg (0700 h)–10 mg (1300 h)–5 mg (1600 h), fludrocortisone 0.1 mg 1–0–0, DHEA 25 mg 1–0–0; long-acting insulin 14E at 2200 h, bolus insulin for meals
Current problems	Nausea and vomiting for 12 h, temperature of 39 °C, diarrhoea
Therapeutic suggestions	Use hydrocortisone emergency kit (i.m. 100 mg hydrocortisone) and call an ambulance for hospital admission
